# A Competing Risk Analysis of Women Dying of Maternal, Infectious, or Non-Communicable Causes in the Kintampo Area of Ghana

**DOI:** 10.3389/fgwh.2021.690870

**Published:** 2021-06-21

**Authors:** Sulemana Watara Abubakari, Delali Margaret Badasu, Edward Anane Apraku, Seeba Amenga-Etego, Kwaku Poku Asante, Ayaga Agula Bawah, Seth Owusu-Agyei

**Affiliations:** ^1^Kintampo Health Research Center, Research and Development Division, Ghana Health Service, Kintampo, Ghana; ^2^Regional Institute for Population Studies, University of Ghana, Accra, Ghana; ^3^Institute of Health Research, University of Health & Allied Sciences, Hohoe, Ghana

**Keywords:** women of reproductive age, maternal, infectious, non-communicable diseases, Kintampo

## Abstract

**Background:** Maternal, infectious, and non-communicable causes of death combinedly are a major health problem for women of reproductive age (WRA) in sub-Saharan Africa (SSA). Little is known about the relative risks of each of these causes of death in their combined form and their demographic impacts. The focus of studies on WRA has been on maternal health. The evolving demographic and health transitions in low- and middle-income countries (LMICs) suggest a need for a comprehensive approach to resolve health challenges of women beyond maternal causes.

**Methods:** Deaths and person-years of exposure (PYE) were calculated by age for WRA within 15–49 years of age in the Kintampo Health and Demographic Surveillance System (KHDSS) area from January 2005 to December 2014. Causes of death were diagnosed using a standard verbal autopsy questionnaire and the 10th Revision of the International Statistical Classification of Diseases and Related Health Problems (ICD-10). Identified causes of death were categorized into three broad areas, namely, maternal, infectious, and non-communicable diseases. Multiple decrements and associated single decrement life table methods were used.

**Results:** Averting any of the causes of death was seen to lead to improved life expectancy, but eliminating infectious causes of death leads to the highest number of years gained. Infectious causes of death affected all ages and the gains in life expectancy, assuming that these causes were eliminated, diminished with increasing age. The oldest age group, 45–49, had the greatest gain in reproductive-aged life expectancy (RALE) if maternal mortality was eliminated.

**Discussion:** This study demonstrated the existence of a triple burden. Infectious causes of death are persistently high while deaths from non-communicable causes are rising and the level of maternal mortality is still unacceptably high. It recommends that attention should be given to all the causes of death among WRA.

## Introduction

Causes of death among women of reproductive age (WRA) are reportedly due to maternal causes and sexual reproductive risks in spite of over three decades of campaign by the WHO and the global health community, in general, to reduce maternal mortality (1, 2). Approximately, 810 women die every day due to maternal-related complications globally and 94% of them occur in low- and middle-income countries (LMICs), especially in sub-Saharan Africa (SSA) (3). The majority of the countries in the SSA region have maternal mortality ratios (MMR) of over 300 maternal deaths per 100,000 live births (4). According to the estimates of WHO, the lifetime risk of maternal death is greatest in SSA, estimated to be 542 (UI 498 to 649), while the lifetime risk of maternal deaths is 1 in 37 and 1 in 7,800 in Australia and New Zealand, respectively (5). Maternal mortality reduced from 870 to 534 maternal deaths per 100,000 live births between 2000 and 2017. Despite the above progress, the SSA region is still lagging behind the European region, with 5 maternal deaths per 100,000 live births in the latter (6).

The general health risks faced by WRA affect maternal mortality (7). Therefore, efforts to reduce maternal mortality should include identifying and addressing other causes of deaths associated with maternal mortality among WRA. However, research and funding have focused mainly on maternal health, while other threats to women of this age-group are neglected (8). Available research evidence suggests that infectious diseases such as HIV/AIDS, TB, and malaria are the leading causes of death across all ages in many LMICs and contribute substantially to deaths among WRA (9). Analysis by Scrafford and Tielsch of 38 countries in three regions, including SSA, showed that deaths from maternal causes contribute between 6 and 40% of all deaths occurring among WRA (1). Besides, some studies have estimated the proportion of infectious diseases among adult deaths to be 40 percent (9), while others have estimated it to be as high as 74% (10). Despite the high contribution of infectious diseases to deaths among WRA and adult mortality, in general, studies on women have focused mainly on maternal health (11, 12).

In addition, studies in recent times have argued for the need to expand maternal health to cover the general health of women and to address the rising causes of death from non-communicable diseases (NCDs) among women. Labrique et al. reported that 48% of deaths among WRA in Bangladesh were due to NCDs (12). Another study in Ethiopia reported that infectious diseases and NCDs were the leading causes of death among women (13). Other studies have also shown that NCDs are going to contribute most to causes of death due to the unfolding demographic and epidemiologic transitions across LMICs, and such studies have emphasized the need to reset priorities on the health of women beyond reproduction (14, 15).

A study by Abubakari et al. that used the Kintampo Health and Demographic Surveillance System (KHDSS) data suggested the need to investigate other causes of death that might be competing with maternal death as far as the population of WRA in the study area is concerned since the evidence from the analysis attests to competition from other causes of death. It was observed that the age patterns of maternal-related mortality and all-cause mortality depicted that maternal-related mortality is different from all-cause mortality for WRA (16).

The rates and numbers of the population or subgroup that die, ages at death, locations of death, and the causes of death are vital for policy direction, planning intervention, and research (17). Yet, in many LMICs, of which Ghana is no exception, information on causes of death is a major challenge to come by. With the ever-increasing challenge posed by the triple burden of causes of death among WRA, where significant causes of death from maternal factors coexist with infectious disease and increasing numbers of NCD causes, there is the need to come up with innovative methods for examining the causes of death within this population. This study used health and demographic surveillance system (HDSS) data that provide empirical evidence in order to examine the relative risks of WRA dying from maternal, infectious, and non-communicable causes among WRA in the Kintampo districts in the Bono East Region of Ghana.

## Materials and Methods

### Study Area and Population

The study area forms part of the catchment area of the Kintampo Health Research Center (KHRC) where the Kintampo HDSS is used. The Kintampo HDSS covers both the Kintampo North Municipality and the Kintampo South District of the Bono East Region of Ghana (18). The two districts are mainly rural, and their district capitals, Kintampo and Jema, and a few other communities in the districts are semi-urban. Together, the two districts as of December 2019 recorded a resident population of 165,816 (19). A detailed description of the Kintampo HDSS operations can be found elsewhere (20).

A total of 1,259 deaths and 329,505 person-years of observation (PYO) were recorded among WRA aged 15–49 years during the 10-year study period (2005–2014) with 162 (12.9%) WRA having no respondents. The two main reasons accounting for the absence of respondents were as follows: (i) difficulty in getting a family member that will be able to provide the required information; and (ii) refusal of a member of the family to either take part in or to complete a verbal autopsy (VA) interview. For some of the VA interviews conducted, there was either insufficient information or specific information was missing for 196 (15.6%) WRA. Because of this challenge, physician coding could not assign any cause of death to such deaths. Accidents contributed 55 (4.4%) cases that were excluded because they were not in the inclusion criteria of this study. The remaining 846 (67.2%) WRA deaths were used as a dataset for analyses in this study.

### Data Collection

The main data collection tool for this study was the Kintampo HDSS women VA questionnaire made up of sections such as background information of the deceased woman, illnesses that led to the death of the woman, and open narration of the circumstances surrounding the death in question by the respondent. Other sections included duration of illnesses or symptoms or whether it was an injury death; specific questions on symptoms and signs during pregnancy, labor, delivery, and 6 weeks after delivery; other illnesses such as heart disease, lung disease, and malaria that are indirect causes of maternal deaths; and medical care sought, as well as socioeconomic, fertility, and obstetric history, and lifestyle of the deceased woman, including tobacco and alcohol use. Data were collected from households where any death of a woman aged from 12 to 50 years old had occurred.

Physician-coded verbal autopsy (PCVA) was used for this study. Two physicians independently reviewed VA questionnaire and assigned a single cause, using the 10th Revision of the International Statistical Classification of Diseases and Related Health Problems (ICD-10). A third clinician was needed to examine the case only when a consensual cause of death is not reached between the first two. No final cause of death is considered when a consensus cannot be reached (21, 22).

### Data Management and Analyses

The VA questionnaires were checked for completeness and consistency by research officers with a minimum of first-degree university education. The VA questionnaires were double-entered on computers using a data management software, Microsoft Visual FoxPro (version 9.0) (23). Automated range and consistency checks were performed. Discrepancies were resolved by referring to the original questionnaire and the field manual that was used for training the field workers.

Life table techniques were employed to examine a situation of competing risks that involve the risk of dying from multiple causes of death. This is based on a hypothetical situation that uses life table methods to estimate by how much life expectancy would increase if any of the causes of death (maternal, infectious, or non-communicable) were eradicated. This is carried out by estimating separately, for each of the causes of death, the expected reduced mortality and the consequent increases in life expectancy that could result in the hypothetical eradication of a specified cause of death (maternal, infectious, or non-communicable). Appendix 1 has a detailed explanation of the procedure.

## Results

### Background Characteristics

A total of 846 WRA, with the age range of 15–49 years, who died between 2005 and 2014 within the Kintampo HDSS were used for this study. The largest and smallest age-groups were within ranges of 30–39 (34.3%) and 15–19 (15.2%) years old, respectively. The majority (63.2%) lived in the rural areas at the time of death, and 57.0% were Christians. More than half (55.9%) had no formal education, and about one out of five attained primary (21.9) or middle school/JHS (22.2%) education. About a third (29.2%) of the deceased participants were Akans, and 17.1% were Gonjas, Dagombas, or Mamprusis. The majority (73.2%) had ever been in a union, more than two out of five (41.3%) were unemployed, and one out of four (25.2%) were in the least poor quintile. More details of the characteristics of deceased participants are shown in Table 1.

**Table 1:** Participant characteristics.

**Background characteristics**	***n*(%)**
**Age-group**	
15–19	129 (15.2)
20–29	234 (27.7)
30–39	290 (34.3)
40–49	193 (22.8)
**Place of residence**	
Rural	534 (63.2)
Urban	312 (36.8)
**Educational level**	
No education	473 (55.9)
Primary	185 (21.9)
Middle/JHS+	188 (22.2)
**Religion**	
Christian	482 (57.0)
Muslim	264 (31.2)
Other	100 (11.8)
**Ethnicity**	
Akan	247 (29.2)
Dagarti, Frafra, Kusasi	113 (13.4)
Gonja, Dagomba, Mamprusi	145 (17.1)
Konkomba, Basare	71 (8.4)
Mo	116 (13.7)
Other	154 (18.2)
**Marital status**	
Ever been in union	619 (73.2)
Never married	227 (26.8)
**Occupation**	
Farmer–laborer–domestic worker	105 (12.4)
Seamstress–hairdresser–trader–food seller	341 (40.3)
Professional–clerical	18 (2.1)
Unemployed	349 (41.3)
Other	33 (3.9)
**Household wealth status**	
Least poor	213 (25.2)
Less poor	176 (20.8)
More poor	158 (18.7)
Most poor	137 (16.2)
Poor	162 (19.1)
Total	846 (100)

### Effects of Eliminating Maternal Causes of Deaths

Table 2 presents a general and multiple-decrement life table for WRA in the Kintampo districts from 2005 to 2014. We restricted the life table to WRA because of our interest in computing reproductive life years saved. For the period 2005–2014, a total of 1,259 deaths occurred among women in their reproductive ages, of which 74 women were estimated to have died from maternal-related causes as defined earlier. In terms of age-group, 116 women died in the 15–19 age-group, 163 in the 20–24 age-group, 213 in the 25–29 age-group, and so on from all-cause mortality. Distribution of the 74 maternal mortality related deaths by age-group were 11 within the 15–19 age-group, 14 in the 20–24 age-group, 25 in the 25–29 age-group, 11 in the 30–34 age-group, 7 in the 35–39 age-group, 4 in the 40–44 age-group, and 2 in the 45–49 age-group. Constructing a single decrement life table for all deaths, irrespective of the cause, results in a life expectancy of 28.7 for women within their reproductive ages (ages 15–49 years).

**Table 2:** A multiple-decrement life table for WRA, Kintampo HDSS (2005–2014).

**Age x**	**PYO**	**D^**All**^**	**D^**MM**^**	**_**n**_a_**x**_**	**_**n**_m_**x**_**	**_**n**_q_**x**_**	**_**n**_p_**x**_**	**l_**x**_**	**_**n**_d_**x**_**	**_**n**_L_**x**_**	**T_**x**_**	**e_**x**_**	**_**n**_ ${\text{q}}_{\text{x}}^{\text{MM}}$ **	**_**n**_d_**x**_^**MM**^**	**l_**x**_^**MM**^**	**_**n**_ ${\text{m}}_{\text{x}}^{\text{MM}}$ **
15–19	70,605	116	11	2.478	0.0016	0.0082	0.9918	100,000	818	497,937	2,865,517	28.7	0.0008	78	703	0.0002
20–24	60,178	163	14	2.711	0.0027	0.0135	0.9865	99,182	1,335	492,853	2,367,580	23.9	0.0012	115	626	0.0002
25–29	52,484	213	25	2.605	0.0041	0.0201	0.9799	97,847	1,966	484,525	1,874,727	19.2	0.0024	231	511	0.0005
30–34	46,333	222	11	2.565	0.0048	0.0237	0.9763	95,881	2,271	473,874	1,390,202	14.5	0.0012	113	280	0.0002
35–39	39,707	227	7	2.534	0.0057	0.0282	0.9718	93,610	2,639	461,544	916,327	9.8	0.0009	81	168	0.0002
40–44	33,456	178	4	2.470	0.0053	0.0262	0.9738	90,971	2,388	448,816	454,784	5.0	0.0006	54	86	0.0001
45–49	26,742	140	2	2.606	0.0052	0.0259	0.9741	88,584	2,290	5,968	5,968	0.1	0.0004	33	33	0.0001
Total	329,505	1,259	74	–	–	–	–	–	–	–	–	–	–	703	–	–

*Source: Kintampo HDSS (2005–2014)*.

*Age x, Age interval; PYO, Person-years of observation; D^All^, All deaths attributable to the cohort; D^MM^, Deaths attributable to maternal causes; _n_a_x_, Average number of person-years lived in the interval by those who have died in the interval; m_x_, Mortality rate for people in age-group x to x + n; _n_q_x_, Probability of dying between ages x and x + n; _n_p_x_, Probability of surviving between ages x and x + n; l_x_, Number surviving at each age; _n_d_x_, Number of deaths between ages x and x + n; _n_L_x_, Person-years lived between ages x and x + n; T_x_, Person-years lived beyond age x; e_x_, Life expectancy at age x; _n_${\text{q}}_{x}^{MM}$, Probability of dying from maternal mortality between ages x and x + n._n_d_x_^MM^, Number of deaths from maternal mortality between ages x and x + n.l_x_^MM^, Number surviving from maternal mortality at each age; _n_${\text{m}}_{x}^{MM}$, Maternal mortality rate for age-group x to x + n*.

To estimate the impact of maternal mortality on overall mortality for women in their reproductive ages, we isolated the 74 deaths due to maternal mortality and estimated a multiple-decrement life table that enabled us to answer the question “*how many women who reached the reproductive age would eventually die from maternal mortality-related causes?”* So, assuming a hypothetical situation where 100,000 women survived from birth to age 15 and were subjected to the age-specific mortality conditions of the period, about 703 of them would eventually die from maternal mortality by the time they reach the age of 49 (Table 2).

Assuming maternal mortality was eliminated, the gains in reproductive-aged life expectancy (RALE) were estimated using caused-deleted life table analysis (Table 3). The resultant RALE is estimated to be 33.1 years. This analysis suggests a gain of 4.4 years during the period 2005–2014 since RALE would have improved from 28.7 to 33.1 years.

**Table 3:** Associated single-decrement life table for causes of death other than maternal causes for Kintampo HDSS from 2005 to 2014.

**Age x**	**l_**x**_**	**_**n**_p_**x**_**	**R^**−MM**^**	**P^**−MM**^**	** ${\text{l}}_{\text{x}}^{-MM}$ **	**_**n**_ ${\text{q}}_{\text{x}}^{-MM}$ **	**_**n**_ ${\text{d}}_{\text{x}}^{-MM}$ **	**_**n**_q_**x**_/_**n**_${\text{q}}_{\text{x}}^{-MM}$**	**_**n**_ ${\text{a}}_{\text{x}}^{-MM}$ **	**_**n**_ ${\text{m}}_{\text{x}}^{-MM}$ **	**_**n**_ ${\text{L}}_{\text{x}}^{-MM}$ **	** ${\text{T}}_{\text{x}}^{-MM}$ **	** ${\text{e}}_{\text{x}}^{-MM}$ **
15–19	100,000	0.9918	0.9052	0.9926	100,000	0.0074	741	1.1043	2.4969	0.0015	498,146	3,306,877	33.1
20–24	99,182	0.9865	0.9141	0.9877	99,259	0.0123	1,222	1.0933	2.6705	0.0025	493,450	2,808,731	28.3
25–29	97,847	0.9799	0.8826	0.9822	98,037	0.0178	1,741	1.1316	2.6133	0.0036	486,031	2,315,282	23.6
30–34	95,881	0.9763	0.9505	0.9775	96,296	0.0225	2,169	1.0515	2.5799	0.0046	476,233	1,829,251	19.0
35–39	93,610	0.9718	0.9692	0.9727	94,128	0.0273	2,573	1.0314	2.5147	0.0055	464,244	1,353,018	14.4
40–44	90,971	0.9738	0.9775	0.9743	91,555	0.0257	2,350	1.0227	2.4892	0.0052	451,875	888,774	9.7
45–49	88,584	0.9741	0.9857	0.9745	89,205	0.0255	2,274	1.0143	0.9857	0.0052	436,899	436,899	4.9

*Source: Kintampo HDSS (2005–2014)*.

*Age x, Age interval; l_x_, Number surviving at each age; _n_p_x_, Probability of surviving between ages x and x + n; R^−MM^, The proportion of deaths due to all causes other than maternal causes; P^−MM^, Probability of surviving all causes of deaths other than maternal; ${\text{l}}_{x}^{-MM}$, Number surviving at each age from all causes of deaths other than maternal; _n_$q_{x}^{-MM}$, Probability of dying from all causes of deaths other than maternal between ages x and x + n; _n_$d_{x}^{-MM}$, Number of deaths from all causes of deaths other than maternal between ages x and x + n; _n_q_x_/ _n_$q_{x}^{-MM}$, Probability of dying between ages x and x + n divided by probability of dying from all causes of deaths other than maternal between ages x and x + n; _n_$a_{x}^{-MM}$, Average number of person-years lived in the interval by those that have died in the interval from all causes other than maternal; _n_$m_{x}^{-MM}$, Mortality rate for people in age-group x to x + n from all causes of deaths other than maternal; _n_$L_{x}^{-MM}$, Person-years lived between ages x and x + n from all causes of deaths other than maternal; $T_{x}^{-MM}$, Person-years lived beyond age x from all causes of deaths other than maternal; $e_{x}^{-MM}$, Life expectancy at age x from all causes of deaths other than maternal*.

### Effects of Eliminating Infectious Causes of Deaths

Table 4 also shows a general and multiple-decrement life table for the whole female population in the Kintampo HDSS area from 2005 to 2014. These estimates result in a life expectancy at birth for the female population at 70.9 years under existing conditions in the Kintampo HDSS area from 2005 to 2014. However, with the general population for the same period, a life expectancy at birth of 66.8 years is recorded (Appendix 2).

**Table 4:** A general and multiple-decrement life table for Kintampo HDSS female population (2005–2014).

**Age x**	**PYO**	**D^**All**^**	**D^**CD**^**	**_**n**_a_**x**_**	**_**n**_m_**x**_**	**_**n**_q_**x**_**	**_**n**_p_**x**_**	**l_**x**_**	**_**n**_d_**x**_**	**_**n**_L_**x**_**	**T_**x**_**	**e_**x**_**	**_**n**_ ${\text{q}}_{\text{x}}^{\text{CD}}$ **	**_**n**_d_**x**_^**CD**^**	**l_**x**_^**CD**^**	**_**n**_ ${\text{m}}_{\text{x}}^{\text{CD}}$ **
<1	20,618	913	386	0.498	0.0443	0.0433	0.9567	100,000	4,332	97,826	7,094,403	70.9	0.0183	1,831	36,107	0.0187
1–4	796,90	512	343	1.746	0.0064	0.0253	0.9747	95,668	2,424	377,211	6,996,577	73.1	0.0170	1,624	34,276	0.0043
5–9	97,717	159	87	2.497	0.0016	0.0081	0.9919	93,245	756	464,054	6,619,366	71.0	0.0044	413	32,652	0.0009
10–14	88,188	124	53	2.483	0.0014	0.0070	0.9930	92,489	648	461,067	6,155,312	66.6	0.0030	277	32,239	0.0006
15–19	70,605	116	38	2.478	0.0016	0.0082	0.9918	91,841	751	458,772	5,694,245	62.0	0.0027	246	31,962	0.0005
20–24	60,178	163	56	2.711	0.0027	0.0135	0.9865	91,090	1,226	454,023	5,235,473	57.5	0.0046	421	31,716	0.0009
25–29	52,484	213	96	2.605	0.0041	0.0201	0.9799	89,864	1,806	445,638	4,781,450	53.2	0.0091	814	31,295	0.0018
30–34	46,333	222	95	2.565	0.0048	0.0237	0.9763	88,058	2,085	436,003	4,335,812	49.2	0.0101	892	30,481	0.0021
35–39	39,707	227	99	2.534	0.0057	0.0282	0.9718	85,973	2,423	423,163	3,899,809	45.4	0.0123	1,057	29,588	0.0025
40–44	33,456	178	77	2.470	0.0053	0.0262	0.9738	83,549	2,193	412,261	3,476,646	41.6	0.0114	949	28,532	0.0023
45–49	26,742	140	58	2.606	0.0052	0.0259	0.9741	81,356	2,103	404,639	3,064,385	37.7	0.0107	871	27,583	0.0022
50–54	21,160	170	63	2.680	0.0080	0.0394	0.9606	79,253	3,125	391,180	2,659,746	33.6	0.0146	1,158	26,712	0.0030
55–59	15,957	173	59	2.621	0.0108	0.0528	0.9472	76,128	4,023	373,278	2,268,566	29.8	0.0180	1,372	25,553	0.0037
60–64	11,914	166	51	2.623	0.0139	0.0674	0.9326	72,105	4,862	353,445	1,895,288	26.3	0.0207	1,494	24,181	0.0043
65–69	8,959	182	56	2.644	0.0203	0.0969	0.9031	67,242	6,518	325,287	1,541,843	22.9	0.0298	2,006	22,688	0.0063
70–74	7,053	206	81	2.613	0.0292	0.1365	0.8635	60,725	8,290	285,929	1,216,556	20.0	0.0537	3,260	20,682	0.0115
75–79	4,949	187	66	2.615	0.0378	0.1733	0.8267	52,434	9,086	241,230	930,627	17.7	0.0612	3,207	17,422	0.0133
80–84	3,311	176	60	2.418	0.0532	0.2337	0.7663	43,348	10,130	342,145	689,397	15.9	0.0797	3,453	14,215	0.0181
85+	4,098	392	127	5.300	0.0957	1.0000	0.0000	33,218	33,218	347,253	347,253	10.5	0.3240	10,762	10,762	0.0310
Total	693,119	4,619	1,951	–	–	–	–	–	–	–	–	–	–	36,107	–	–

*Source: Kintampo HDSS (2005–2014)*.

*Age x, Age interval; PYO, Person-years of observation; D^All^ _= All deaths attributable to the cohort; D^CD^_= Deaths attributable to communicable disease; _n_a_x_, Average number of person-years lived in the interval by those that have died in the interval; _n_m_x_, Mortality rate for people in age-group x to x + n; _n_q_x_, Probability of dying between ages x and x + n; _n_p_x_, Probability of surviving between ages x and x + n; l_x_, Number surviving at each age; _n_d_x_, Number of deaths between ages x and x + n; _n_L_x_, Person-years lived between ages x and x + n; T_x_, Person-years lived beyond age x; e_x_, Life expectancy at age; x._n_$q_{\text{x}}^{\text{CD}}$, Probability of dying from communicable disease between ages x and x + n; _n_d_x_^CD^, Number of deaths from communicable disease between ages x and x + n; l_x_^CD^, Number surviving from communicable disease at each age; _n_$m_{\text{x}}^{\text{CD}}$, Mortality rate for communicable disease from age-group x to x + n*.

The age-specific mortality rate (ASMR) for 15–19 years old (_5_*m*_15_) is estimated to be 0.002, and the corresponding probability of dying between ages 15 and 19 for those that survive till age 15 (_5_*q*_15_) is 0.008, resulting in a life expectancy of 62 years at age 15 for the female population in the Kintampo HDSS area from 2005 to 2014. Similarly, the ASMR for 45–49 years old (_5_*m*_45_) is estimated to be 0.005, and the corresponding probability of dying between ages 45 and 49 for those that survive to age 45 (_5_*q*_45_) is 0.026, resulting in a life expectancy at age 45 of 37.7 years for the female population in the Kintampo districts between 2005 and 2014.

With a cohort of 100,000 female newborns, it is expected that 36,107 of them may ultimately die of infectious-related mortality by age 85, supposing that the mortality conditions of 2005–2014 persist during their life span. This suggests that over 36% of all female newborns may ultimately die of infectious diseases by age 85. In the case of WRA, starting at age 15 with a cohort of 91,841 WRA (15–49), 5,254 of them are estimated to eventually die of infectious disease-related mortality by the time they are 49 years old, if the age-specific mortality conditions of 2005–2014 persisted for the ages 15–49. This means that about 5.7% of WRA (15–49) may ultimately die of infectious diseases by the time they are 49 years old.

**Figure 1:**
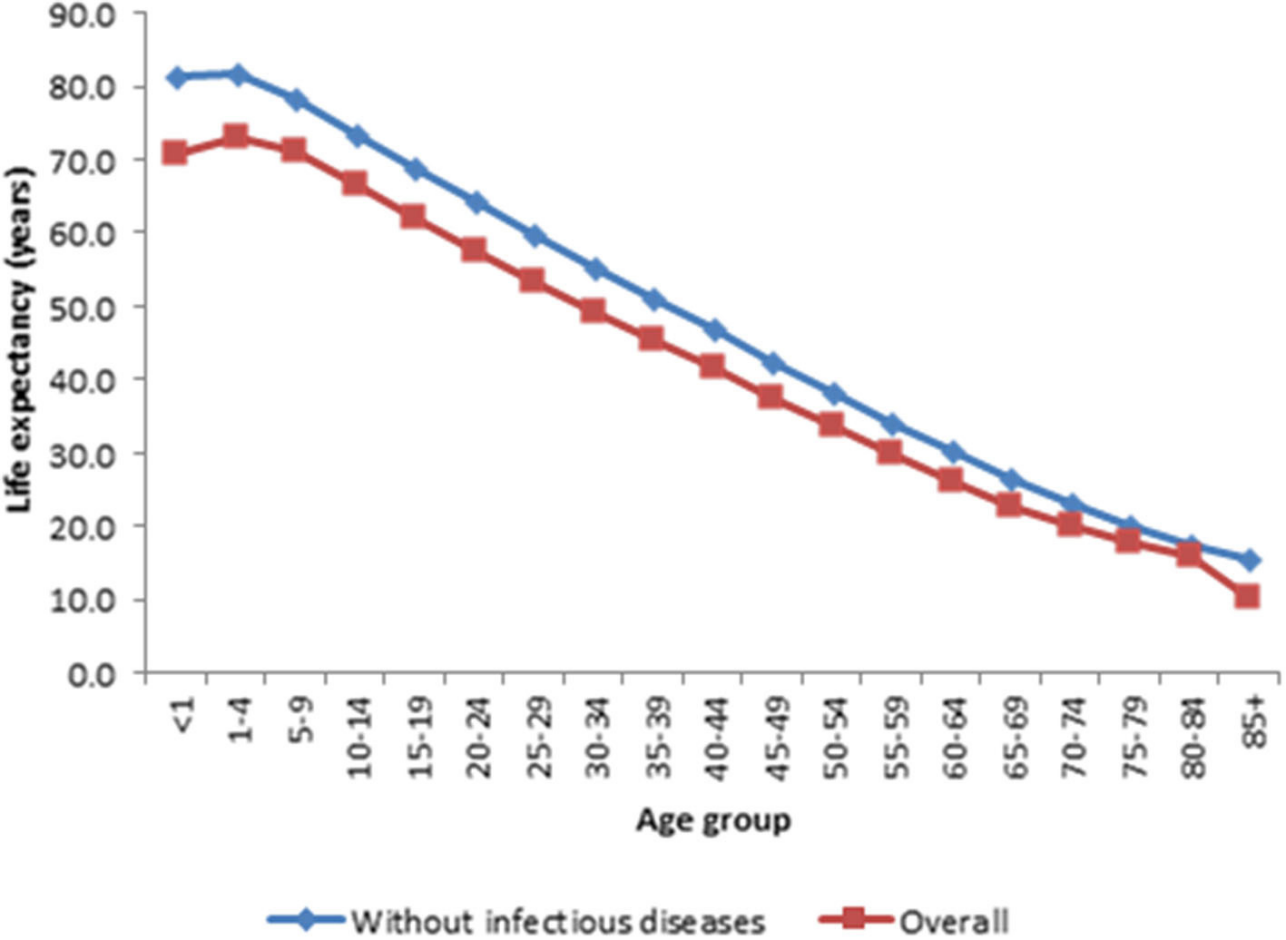
Comparsion of estimated life expectancy for each age group with all cause of death and without infection diseases, Kintampo HDSS (2005–2014). Source: Kintampo HDSS (2005–2014).

Figure 1 presents life expectancy at every age when all the causes of death were pooled together and the corresponding life expectancies when infectious causes were removed. The results shown in Figure 1 demonstrate that life expectancy improved at each age if infectious causes of death were averted. As expected, the rise in life expectancy is more noticeable at the earlier ages of life compared to other ages. More details are provided in Appendix 3.

### Effects of Eliminating NCD Causes of Deaths

**Figure 2:**
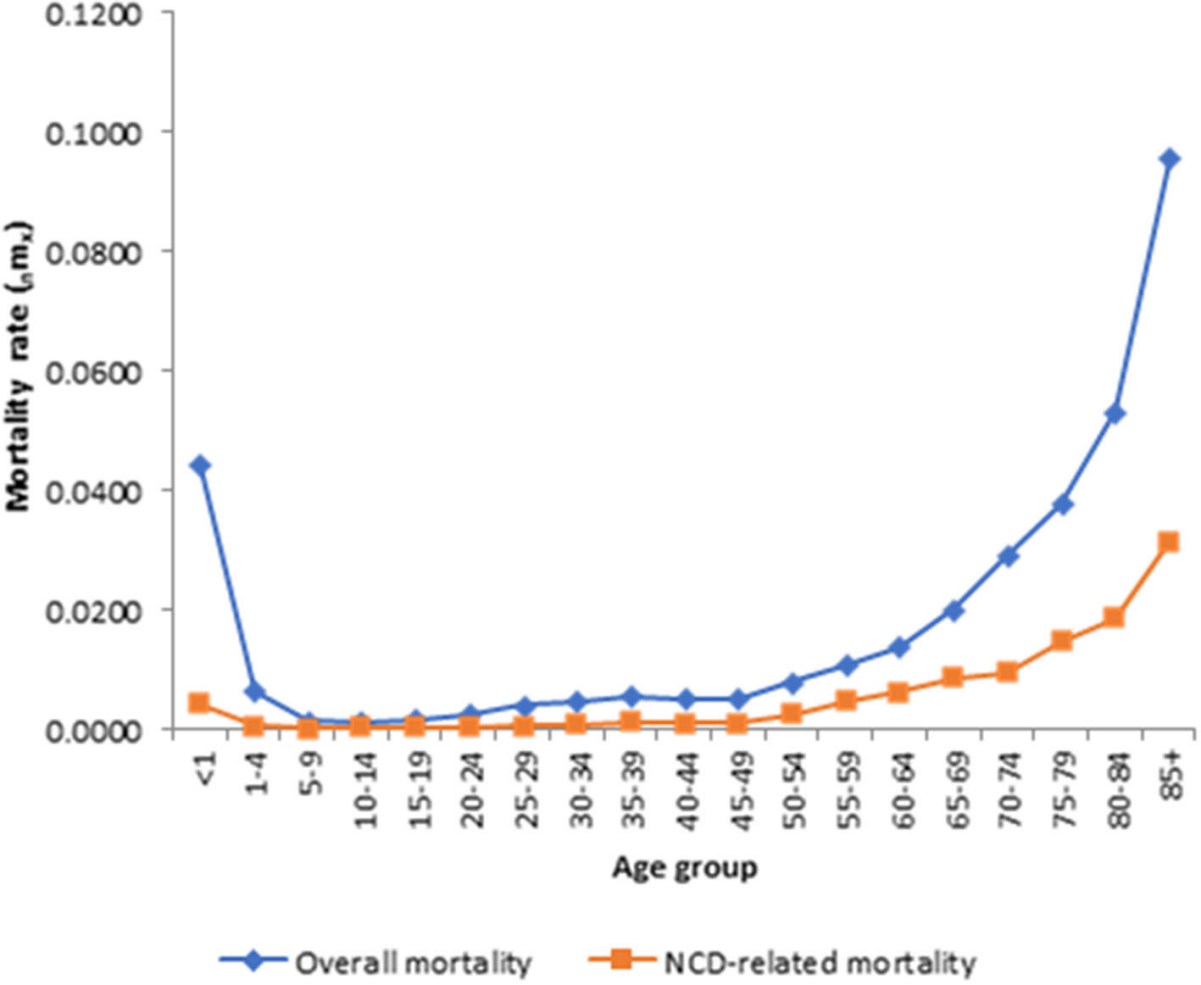
Age pattern of infectious diseases for overall morality and infections causes-specific mortality. Source: Kintampo HDSS (2005–2014).

Figure 2 shows the age pattern of causes of death for overall mortality and NCD-related mortality. The results demonstrate that the age pattern of mortality for NCD-related mortality is different from that of overall mortality. Relatively, mortality rates are high after the ages of 49 years for the overall mortality and 69 years for the NCD-related mortality but very low before age 5. Mortality rates also are generally much higher after age 60. This suggests that the level of susceptibility to the causes of death and to NCD-related mortality, in particular, increased considerably after 69 years of age. Further details are provided in Appendix 4.

**Figure 3:**
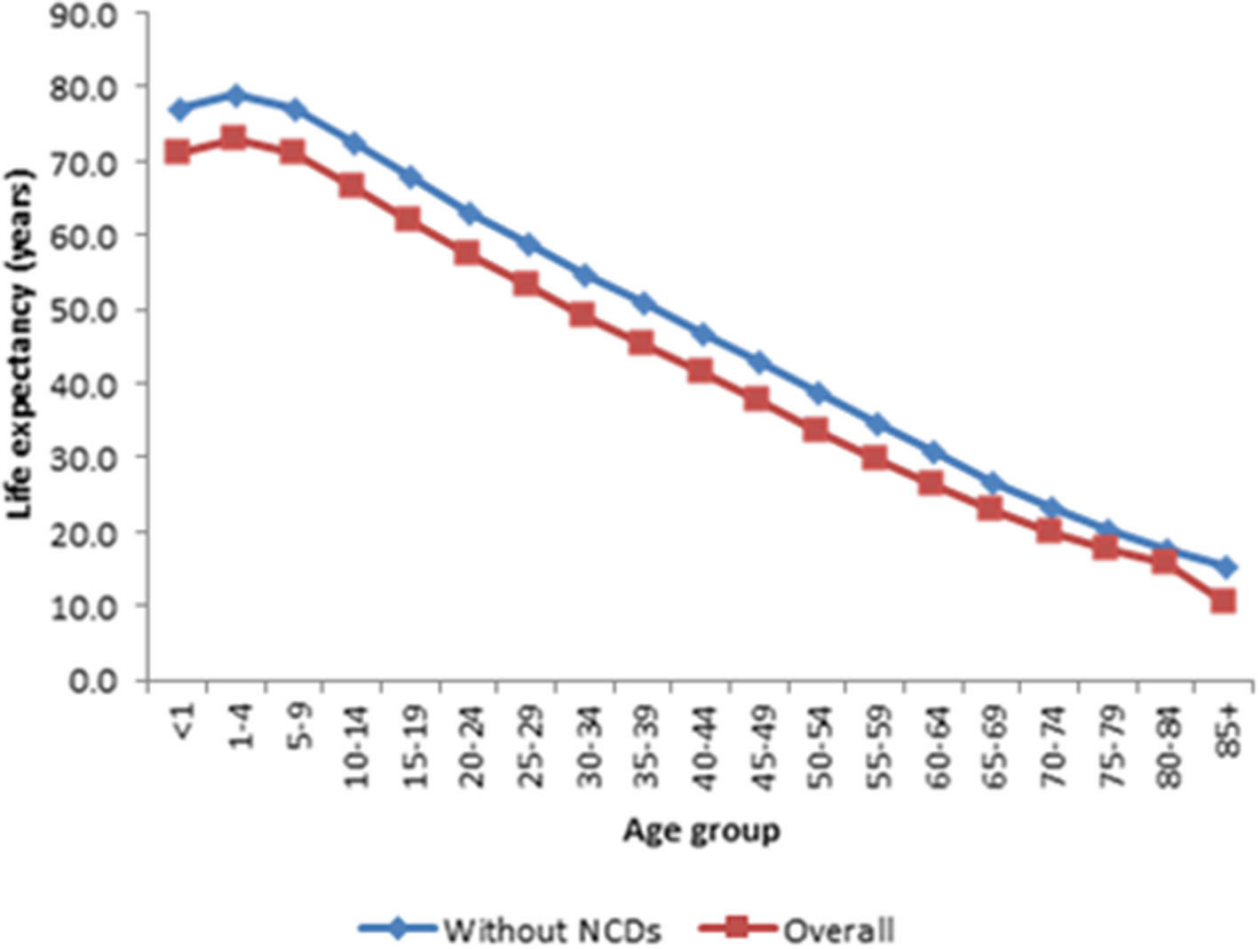
Comparison of estimated life expectancy for each age group with all cause of death and without non-communicable diseases, Kintampo HDSS (2005–2014). Source: Kintampo HDSS (2005–2014).

Figure 3 presents life expectancy at every age when all causes of death were pooled together and the corresponding life expectancies when non-communicable diseases (NCDs) were averted. The results depicted in the figure show that life expectancy increased at every age in the absence of NCD deaths. As expected, the increase in life expectancy is less pronounced at the early ages of life compared to the pattern in Figure 1. This observation is because NCDs disproportionately affect the older population. Other details are provided in Appendix 5.

## Discussion

This study estimated the potential gains in life expectancy, assuming that maternal, infectious, or non-communicable causes of death were averted. It was observed that eradicating any of the causes of death considered leads to improved life expectancy. However, some of the causes of death contributed more significantly compared to others. The gains in life expectancy after the eradication of any cause of death were different for the various age-groups. Similar to the observations in this study, Arriaga (24) and De Castro (25) reported that when comparing abridged life tables with different mortality levels, mortality differs by different magnitude in all age-groups is observed in most cases.

The study suggests that eradicating infectious causes of death leads to the highest number of years gained in life expectancy. Eradication of infectious disease-related deaths increased life expectancy at age 15 by 6.8 years, an impact that is much greater compared to NCD (5.9 years) and maternal mortality (4.4 years).

This finding also suggests that mortality in the study area is at the first stage of the epidemiologic transition with infectious diseases as the main cause of mortality (26). According to the epidemiological transition theory, this is because deaths from cardiovascular, neoplasm, and other NCD conditions increasingly become the main causes when deaths from infectious causes are reduced to very low levels (26). However, the situation in the study area could be best described as a “protracted polarized model” (27) since there is an increased risk of both infectious and non-communicable relative to maternal causes of death. This observation from the present study suggests that both infectious and non-communicable causes of death predominantly coexist among WRA in the two Kintampo districts.

In addition, it was observed that the burden of infectious causes of death was heaviest among WRA relative to non-communicable and maternal causes of death. This observation is further corroborated by the fact that from age 15, about 5.7% of WRA (15–49) may ultimately die of infectious diseases by age 49. In contrast, about half of this proportion who may finally die of NCDs by age 49 and approximately one-eighth of the proportion who may finally die of infectious causes of death could in the end die of maternal causes of death by age 49.

The findings of the present study are consistent with the nature of the causes of death found in low-income settings. It has been observed that the greatest gain in life expectancy across high-income countries occurred from the eradication of non-communicable causes of death, whereas most of the large gains in life expectancy in LMICs happened from the elimination of causes of death due to infectious diseases (28). Furthermore, a study in Pakistan that examined the gains in life expectancy after elimination of specific causes of death reported that the elimination of causes of death due to circulatory system diseases resulted in a lower gain in life expectancy of 1.29 years compared to 3.9 years gained if malaria were eliminated (29). The finding of the Pakistani study is similar to that of this study, where removal of infectious causes of death resulted in the highest gain in life expectancy. Yet, in a more recent study in another Asian country with relatively higher income levels, cardiovascular diseases (CVDs) contributed the highest number of years gained in life expectancy (30). The foregoing discussions suggest that causes of death are context and time specific.

Moreover, Canudas-Romo et al. (31) used the demographic and health survey and other data sources to estimate the improvement in RALE in developed and African countries by eliminating maternal mortality. Their study involving data from 28 SSA countries documented RALE values ranging from 27.9 to 33.4 years across these countries. The results of the present study indicated a RALE of 28.7 years, which is within the range estimated by Canudas-Romo et al. (31) for SSA countries.

Causes of death may not have been estimated accurately since not all deaths recorded by the Kintampo HDSS had successful VA interviews, but this is expected to be self-selected and, therefore, should not have major effects on this study. In addition, a proportion of the cases with successful interviews were coded as “cause of death not determined.” This is also expected to be random. Furthermore, there are possibilities of wrongly assigning causes of deaths but, again, this is not expected to significantly affect the results since they are very likely to be self-selected. Moreover, child deaths are more likely to be missed than adult deaths. As a cultural practice, infants that die a few days or weeks after delivery are usually not reported by some family members that do not count them as human beings. Individuals considered for this study were all adults that were less likely to have been missed under such circumstances.

## Conclusions

This study has demonstrated the existence of a triple burden of maternal, infectious, and non-communicable causes of death among the WRA in the two districts of Kintampo. Infectious causes of death were persistently high. Deaths from non-communicable causes were seen to be on the rise over the years with the levels of maternal mortality being unacceptably high. This highlights the need to pay equal attention to all the causes of death among women of WRA.

## Data Availability Statement

The raw data supporting the conclusions of this article will be made available by the authors, without undue reservation.

## Ethics Statement

The studies involving human participants were reviewed and approved by Kintampo Health Research Institutional Ethics committee. Written informed consent to participate in this study was provided by the participants' legal guardian/next of kin.

## Author Contributions

SA drafted the manuscript. SA, AB, and DB designed the study. SA, EA, SA-E, KA, and SO-A conducted the study. SA, AB, DB, KA, and SO-A participated in the statistical analyses, interpretation, and manuscript revisions. All the authors approved the final version and agreed to be accountable for the study.

## Conflict of Interest

The authors declare that the research was conducted in the absence of any commercial or financial relationships that could be construed as a potential conflict of interest.

## Abbreviations

ASMR: Age-specific mortality rate
CVDs: cardiovascular diseases
HDSS: Health and Demographic Surveillance System
HIV/AIDS: human immunodeficiency virus/acquired immunodeficiency syndrome
ICD-10: International Statistical Classification of Disease and Related Health Problems Version 10
KHDSS: Kintampo Health and Demographic Surveillance System
KHRC: Kintampo Health Research Center
LMICs: low- and middle-income countries
MMR: maternal mortality ratio
MNCH: Maternal, Neonatal, and Child Health
NCDs: non-communicable diseases
PCVA: physician-coded verbal autopsy
PYO: person-years of observation
RALE: reproductive-aged life expectancy
RIPS: Regional Institute for Population Studies
SSA: sub-Saharan Africa
VA: verbal autopsy
WHO: World Health Organization
WNCH: Women, Neonatal and Child Health
WRA: women of reproductive age.
