# Supplementary material for: A Competing Risk Analysis of Women Dying of Maternal, Infectious, or Non-Communicable Causes in the Kintampo Area of Ghana
Source: Front Glob Womens Health. 2021 Jun 21;2:690870. doi: 10.3389/fgwh.2021.690870 (PMC8593997; doi:10.3389/fgwh.2021.690870)
Supplement: Supplementary file 1 [file Data_Sheet_1.docx]

**Appendix I**

**Estimation Procedure for Multiple Decrement and Associated Single Decrement**

**Life Tables**

In the analysis of causes of death, the force of the mortality function from different causes is additive because disentangling precisely the effects of other causes of death is difficult, especially in settings where precise measurement is not possible. Thus, the sum of the different causes is equal to all causes combined as represented in equation (1) thus:


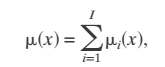
 (1)

where µ(*x*) is the force of mortality from all causes combined and parameters µ*_i_*(*x*) refer to the death rate for the *i*th cause of death. This implies that the rates of decrements are also additive:


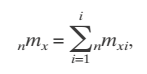
 (2)

where *_n_m_x_* is the rate of decrement from all causes and *_n_m_xi_* in this case is the rate of decrement from maternal or infectious or non-communicable causes of death.

Considering the basic relationship between mortality rates (*_n_m_x_*) and the probability of dying (*_n_q_x_*) as shown in the conventional life table, the transformation of the rates to probabilities of dying is shown in the following equation as:


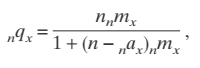
 (3)

where *_n_a_x_* is defined as the average number of person-years lived in the interval *x* to *x + n* by those who died in the interval. This relationship extends to multiple-decrement processes as follows:


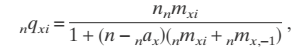
 (4)

where *_n_m_xi_* and *_n_m_x,–i_* represent decrement rates from maternal or infectious or non-communicable and all other causes other than maternal or infectious or non-communicable combined, respectively. Data concerning the causes of death by age and the corresponding number of person-years by the same sub-categories define the probabilities of dying at each age (*_n_q_x_*), by cause of death. However, obtaining the *_n_a_x_* values is often difficult. Therefore, different techniques are employed to estimate the *_n_a_x_* values. First, it is assumed that those who died in the interval on average lived halfway through the interval. Based on this assumption, an initial value of 2.5 is adopted for all age groups with an interval of 5 years. For the younger than 1-year and 1–4-year age groups, a procedure suggested by Coale and Demeny is adopted (Bawah and Binka, 2007).

Using the *_n_a_x_* values of 2.5 in the *_n_m_x_*→ *_n_q_x_* conversion formula, *_n_q_x_* values are estimated first and the values are used to obtain *ndx* (the number of deaths between age *x* and *x + n*) in a life table. These *_n_d_x_* estimates are plugged into the iteration formula below to obtain new sets of *_n_a_x_* values. These values are subsequently re-introduced into the *_n_m_x_*→ *_n_q_x_* conversion formula to re-estimate new *_n_d_x_* values, which are re-introduced in the iteration formula to obtain a new set of *_n_a_x_* values. This process is repeated until stable estimates of *_n_a_x_* are achieved (Bawah and Binka, 2007). The iteration equation used is specified as follows:


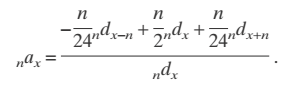
 (5)

The stable *_n_a_x_* values then are used to generate a life table for females in the Kintampo HDSS area through the basic *_n_m^x^* → *_n_q_x_* conversion formula. With the overall life table generated, the probability of dying from maternal, infectious or non-communicable causes of death (*_n_q_xi_*) is estimated, by applying the proportion of deaths that are due to maternal, infectious or non-communicable causes of death to the overall probabilities of dying for each age, *_n_q_x_*, as follows:


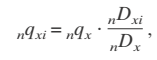
 (6)

where *_n_q_xi_* and *_n_D_xi_* represent the probability of dying from maternal, infectious or non-communicable causes of death and the observed number of deaths from maternal, infectious or non-communicable causes of death, respectively. The above relationship assumes that the observed death rates for maternal, infectious or non-communicable causes of death (*_n_M_xi_*) are equal to the life-table death rates for maternal, infectious or non-communicable causes of death (*_n_m_xi_*), that is, *_n_M_xi_ = _n_m_xi_*.

Estimating the contribution of mortality from maternal, infectious or non-communicable causes of death to overall mortality also allow to estimate the effect of eliminating maternal, infectious or non-communicable causes of death through “cause-deleted” lifetable analysis (Bawah and Binka, 2007). If maternal, infectious or non-communicable causes-related mortality were eliminated as a cause of death, survival at age interval *x* to *x* + *n*, will be represented as:


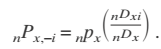
 (7)

The approach described above assumes that the force of mortality function from each cause is proportional to all causes combined in the interval *x* to *x + n* and constant throughout the interval (Arriaga, 1984). The *_n_a_x_* values for the associated single decrement life table were obtained using the following formula for all age groups except the first two and the last:


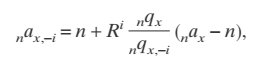
 (8)

where *_n_a_x_, _−i_* refers to the average number of person-years lived by those dying in the interval from all causes other than maternal, infectious or non-communicable-related death, and *Ri* represents the proportion of deaths due to maternal, infectious or non-communicable-related mortality. For the other age groups, the iteration procedure used for estimating the *_n_a_x_* values in the parent life table is used.
