# Supplementary material for: A Competing Risk Analysis of Women Dying of Maternal, Infectious, or Non-Communicable Causes in the Kintampo Area of Ghana
Source: Front Glob Womens Health. 2021 Jun 21;2:690870. doi: 10.3389/fgwh.2021.690870 (PMC8593997; doi:10.3389/fgwh.2021.690870)
Supplement: Supplementary file 2 [file Data_Sheet_2.docx]

**Appendix II: A general life table for Kintampo HDSS (2005 to 2014)**

| Age x | PYO | D^All^ | _n_a_x_ | _n_m_x_ | _n_q_x_ | _n_p_x_ | l_x_ | _n_d_x_ | _n_L_x_ | T_x_ | e_x_ |
| --- | --- | --- | --- | --- | --- | --- | --- | --- | --- | --- | --- |
| <1 | 41,880 | 2065 | 0.4981 | 0.0493 | 0.0481 | 0.9519 | 100,000 | 4,812 | 97,585 | 6,675,182 | 66.8 |
| 1-4 | 161,275 | 1065 | 1.7463 | 0.0066 | 0.0260 | 0.9740 | 95,188 | 2,477 | 375,170 | 6,577,597 | 69.1 |
| 5-9 | 194,939 | 353 | 2.4972 | 0.0018 | 0.0090 | 0.9910 | 92,711 | 836 | 461,083 | 6,202,427 | 66.9 |
| 10-14 | 181,753 | 273 | 2.4830 | 0.0015 | 0.0075 | 0.9925 | 91,875 | 687 | 458,067 | 5,741,344 | 62.5 |
| 15-19 | 150,089 | 284 | 2.4777 | 0.0019 | 0.0094 | 0.9906 | 91,188 | 859 | 455,216 | 5,283,277 | 57.9 |
| 20-24 | 116,138 | 341 | 2.7105 | 0.0029 | 0.0146 | 0.9854 | 90,329 | 1,317 | 449,909 | 4,828,061 | 53.4 |
| 25-29 | 93,259 | 394 | 2.6049 | 0.0042 | 0.0209 | 0.9791 | 89,012 | 1,861 | 441,413 | 4,378,152 | 49.2 |
| 30-34 | 82,110 | 421 | 2.5651 | 0.0051 | 0.0253 | 0.9747 | 87,151 | 2,207 | 432,140 | 3,936,739 | 45.2 |
| 35-39 | 72,854 | 511 | 2.5340 | 0.0070 | 0.0345 | 0.9655 | 84,944 | 2,928 | 417,597 | 3,504,599 | 41.3 |
| 40-44 | 62,648 | 474 | 2.4701 | 0.0076 | 0.0371 | 0.9629 | 82,015 | 3,044 | 402,951 | 3,087,002 | 37.6 |
| 45-49 | 50,880 | 408 | 2.6058 | 0.0080 | 0.0393 | 0.9607 | 78,971 | 3,107 | 390,129 | 2,684,051 | 34.0 |
| 50-54 | 42,104 | 459 | 2.6797 | 0.0109 | 0.0532 | 0.9468 | 75,864 | 4,033 | 373,066 | 2,293,922 | 30.2 |
| 55-59 | 33,033 | 506 | 2.6205 | 0.0153 | 0.0739 | 0.9261 | 71,831 | 5,308 | 348,269 | 1,920,855 | 26.7 |

**(Continued) A general life table for Kintampo HDSS (2005 to 2014)**

| 60-64 | 25,028 | 469 | 2.6233 | 0.0187 | 0.0897 | 0.9103 | 66,523 | 5,967 | 322,950 | 1,572,586 | 23.6 |
| --- | --- | --- | --- | --- | --- | --- | --- | --- | --- | --- | --- |
| 65-69 | 18,293 | 490 | 2.6438 | 0.0268 | 0.1260 | 0.8740 | 60,556 | 7,629 | 286,664 | 1,249,637 | 20.6 |
| 70-74 | 13,981 | 482 | 2.6132 | 0.0345 | 0.1593 | 0.8407 | 52,927 | 8,430 | 246,092 | 962,973 | 18.2 |
| 75-79 | 9,548 | 429 | 2.6146 | 0.0449 | 0.2029 | 0.7971 | 44,497 | 9,029 | 201,093 | 716,881 | 16.1 |
| 80-84 | 6,327 | 409 | 2.4176 | 0.0646 | 0.2770 | 0.7230 | 35,468 | 9,824 | 264,130 | 515,788 | 14.5 |
| 85+ | 7,399 | 754 | 5.3000 | 0.1019 | 1.0000 | 0.0000 | 25,644 | 25,644 | 251,657 | 251,657 | 9.8 |
| Total | 1,363,537 | 10587 |  |  |  |  |  |  |  |  |  |
