# Supplementary material for: A Competing Risk Analysis of Women Dying of Maternal, Infectious, or Non-Communicable Causes in the Kintampo Area of Ghana
Source: Front Glob Womens Health. 2021 Jun 21;2:690870. doi: 10.3389/fgwh.2021.690870 (PMC8593997; doi:10.3389/fgwh.2021.690870)
Supplement: Supplementary file 3 [file Data_Sheet_3.docx]

**Appendix III: Associated single-decrement life table for causes of death other than infectious diseases for Kintampo HDSS from 2005 to 2014**

| Age x | l_x_ | _n_p_x_ | R^-CD^ | P^-CD^ | l_x_^-CD^ | _n_q_x_^-CD^ | _n_d_x_^-CD^ | _n_q_x_/ _n_q_x_^-CD^ | _n_a_x_^-CD^ | _n_m_x_^-CD^ | _n_L_x_^-CD^ | T_x_^-CD^ | e_x_^-CD^ |
| --- | --- | --- | --- | --- | --- | --- | --- | --- | --- | --- | --- | --- | --- |
| <1 | 100000 | 0.9567 | 0.5772 | 0.9748 | 100000 | 0.0252 | 2524 | 1.7164 | 0.4979 | 0.0256 | 98733 | 8146843 | 81.5 |
| 1-4 | 95668 | 0.9747 | 0.3301 | 0.9916 | 98673 | 0.0084 | 832 | 3.0037 | 2.0666 | 0.0021 | 393085 | 8048110 | 81.6 |
| 5-9 | 93245 | 0.9919 | 0.4528 | 0.9963 | 97841 | 0.0037 | 360 | 2.2034 | 2.4985 | 0.0007 | 488306 | 7655025 | 78.2 |
| 10-14 | 92489 | 0.9930 | 0.5726 | 0.9960 | 97481 | 0.0040 | 392 | 1.7439 | 2.5931 | 0.0008 | 486465 | 7166719 | 73.5 |
| 15-19 | 91841 | 0.9918 | 0.6724 | 0.9945 | 97090 | 0.0055 | 535 | 1.4852 | 2.6806 | 0.0011 | 484209 | 6680254 | 68.8 |
| 20-24 | 91090 | 0.9865 | 0.6564 | 0.9911 | 96555 | 0.0089 | 855 | 1.5198 | 2.6198 | 0.0018 | 480740 | 6196045 | 64.2 |
| 25-29 | 89864 | 0.9806 | 0.5493 | 0.9893 | 95700 | 0.0107 | 1027 | 1.8735 | 2.5869 | 0.0022 | 476023 | 5715305 | 59.7 |
| 30-34 | 88117 | 0.9764 | 0.5721 | 0.9864 | 94673 | 0.0136 | 1283 | 1.7469 | 2.5779 | 0.0027 | 470259 | 5239282 | 55.3 |
| 35-39 | 86039 | 0.9716 | 0.5639 | 0.9839 | 93390 | 0.0161 | 1507 | 1.7472 | 2.5107 | 0.0032 | 463200 | 4769023 | 51.1 |
| 40-44 | 83593 | 0.9740 | 0.5674 | 0.9852 | 91883 | 0.0148 | 1361 | 1.7721 | 2.4788 | 0.0030 | 455986 | 4305823 | 46.9 |
| 45-49 | 81423 | 0.9743 | 0.5857 | 0.9849 | 90522 | 0.0151 | 1368 | 1.7102 | 2.6322 | 0.0031 | 449372 | 3849838 | 42.5 |
| 50-54 | 79333 | 0.9606 | 0.6294 | 0.9750 | 89154 | 0.0250 | 2229 | 1.5770 | 2.6576 | 0.0051 | 440548 | 3400466 | 38.1 |
| 55-59 | 76204 | 0.9472 | 0.6590 | 0.9649 | 86925 | 0.0351 | 3055 | 1.5037 | 2.6180 | 0.0071 | 427347 | 2959917 | 34.1 |
| 60-64 | 72177 | 0.9326 | 0.6928 | 0.9528 | 83870 | 0.0472 | 3960 | 1.4282 | 2.6258 | 0.0097 | 409948 | 2532571 | 30.2 |
| 65-69 | 67310 | 0.9031 | 0.6923 | 0.9318 | 79910 | 0.0682 | 5446 | 1.4223 | 2.5913 | 0.0141 | 386432 | 2122623 | 26.6 |
| 70-74 | 60786 | 0.8635 | 0.6068 | 0.9148 | 74464 | 0.0852 | 6346 | 1.6020 | 2.5803 | 0.0177 | 356965 | 1736190 | 23.3 |
| 75-79 | 52487 | 0.8267 | 0.6471 | 0.8841 | 68118 | 0.1159 | 7892 | 1.4957 | 2.5883 | 0.0244 | 321558 | 1379225 | 20.2 |
| 80-84 | 43392 | 0.7663 | 0.6591 | 0.8391 | 60226 | 0.1609 | 9691 | 1.4524 | 2.4271 | 0.0350 | 276198 | 1057667 | 17.6 |
| 85+ | 33251 | 0.0000 | 0.6760 | 0.0000 | 50536 | 1.0000 | 50536 | 1.0000 | 15.4637 | 0.0647 | 781469 | 781469 | 15.5 |

**Source: Kintampo HDSS (2005-2014)**

**Note**:

Age x = Age interval.

l_x_ = Number surviving at each age.

_n_p_x_ = Probability of surviving between ages x and x + n.

R^− CD^ = the proportion of deaths due to all causes other than communicable diseases.

P^- CD^ = Probability of surviving all causes of deaths other than communicable diseases.

l_x_^- CD^ = Number surviving at each age from all causes of deaths other than communicable diseases.

_n_q_x_^- CD^ = Probability of dying from all causes of deaths other than communicable diseases between ages x and x + n.

_n_d_x_^- CD^ = Number of deaths from all causes of deaths other than communicable diseases between ages x and x + n.

_n_q_x_/ _n_q_x_^- CD^ = Probability of dying between ages x and x + n divided by probability of dying from all causes of deaths other than communicable diseases between ages x and x + n.

_n_a_x_^- CD^ = Average number of person-years lived in the interval by those who have died in the interval from all causes other than communicable diseases.

_n_m_x_^- CD^ = Mortality rate for people in age group x to x + n from all causes of deaths other than communicable diseases.

_n_L_x_^- CD^ = Person-years lived between ages x and x + n from all causes of deaths other than communicable diseases.

T_x_^- CD^ = Person-years lived beyond age x from all causes of deaths other than communicable diseases.

e_x_^- CD^ = Life expectancy at age x from all causes of deaths other than communicable diseases.
