# Supplementary material for: A Competing Risk Analysis of Women Dying of Maternal, Infectious, or Non-Communicable Causes in the Kintampo Area of Ghana
Source: Front Glob Womens Health. 2021 Jun 21;2:690870. doi: 10.3389/fgwh.2021.690870 (PMC8593997; doi:10.3389/fgwh.2021.690870)
Supplement: Supplementary file 4 [file Data_Sheet_4.docx]

**Appendix IV: A general and a multiple-decrement life table for Kintampo HDSS female population (2005-2014)**

| Age x | PYO | D^All^ | D^NCD^ | _n_a_x_ | _n_m_x_ | _n_q_x_ | _n_p_x_ | l_x_ | _n_d_x_ | _n_L_x_ | T_x_ | e_x_ | _n_q_x_^NCD^ | _n_d_x_ ^NCD^ | l_x_ ^NCD^ | _n_m_x_^NCD^ |
| --- | --- | --- | --- | --- | --- | --- | --- | --- | --- | --- | --- | --- | --- | --- | --- | --- |
| <1 | 20618 | 913 | 91 | 0.498 | 0.0443 | 0.0433 | 0.9567 | 100000 | 4332 | 97826 | 7094403 | 70.9 | 0.0043 | 432 | 32031 | 0.0044 |
|  |  |  |  |  |  |  |  |  |  |  |  |  |  |  |  |  |
| 1-4 | 79690 | 512 | 51 | 1.746 | 0.0064 | 0.0253 | 0.9747 | 95668 | 2424 | 377211 | 6996577 | 73.1 | 0.0025 | 241 | 31599 | 0.0006 |
|  |  |  |  |  |  |  |  |  |  |  |  |  |  |  |  |  |
| 5-9 | 97717 | 159 | 22 | 2.497 | 0.0016 | 0.0081 | 0.9919 | 93245 | 756 | 464054 | 6619366 | 71.0 | 0.0011 | 105 | 31358 | 0.0002 |
|  |  |  |  |  |  |  |  |  |  |  |  |  |  |  |  |  |
| 10-14 | 88188 | 124 | 40 | 2.483 | 0.0014 | 0.0070 | 0.9930 | 92489 | 648 | 461067 | 6155312 | 66.6 | 0.0023 | 209 | 31253 | 0.0005 |
|  |  |  |  |  |  |  |  |  |  |  |  |  |  |  |  |  |
| 15-19 | 70605 | 116 | 27 | 2.478 | 0.0016 | 0.0082 | 0.9918 | 91841 | 751 | 458772 | 5694245 | 62.0 | 0.0019 | 175 | 31044 | 0.0004 |
|  |  |  |  |  |  |  |  |  |  |  |  |  |  |  |  |  |
| 20-24 | 60178 | 163 | 32 | 2.711 | 0.0027 | 0.0135 | 0.9865 | 91090 | 1226 | 454023 | 5235473 | 57.5 | 0.0026 | 241 | 30869 | 0.0005 |
|  |  |  |  |  |  |  |  |  |  |  |  |  |  |  |  |  |
| 25-29 | 52484 | 213 | 36 | 2.605 | 0.0041 | 0.0201 | 0.9799 | 89864 | 1806 | 445638 | 4781450 | 53.2 | 0.0034 | 305 | 30629 | 0.0007 |
|  |  |  |  |  |  |  |  |  |  |  |  |  |  |  |  |  |
| 30-34 | 46333 | 222 | 42 | 2.565 | 0.0048 | 0.0237 | 0.9763 | 88058 | 2085 | 436003 | 4335812 | 49.2 | 0.0045 | 395 | 30324 | 0.0009 |
|  |  |  |  |  |  |  |  |  |  |  |  |  |  |  |  |  |
| 35-39 | 39707 | 227 | 50 | 2.534 | 0.0057 | 0.0282 | 0.9718 | 85973 | 2423 | 423163 | 3899809 | 45.4 | 0.0062 | 534 | 29929 | 0.0013 |
|  |  |  |  |  |  |  |  |  |  |  |  |  |  |  |  |  |
| 40-44 | 33456 | 178 | 36 | 2.470 | 0.0053 | 0.0262 | 0.9738 | 83549 | 2193 | 412261 | 3476646 | 41.6 | 0.0053 | 444 | 29395 | 0.0011 |
|  |  |  |  |  |  |  |  |  |  |  |  |  |  |  |  |  |
| 45-49 | 26742 | 140 | 30 | 2.606 | 0.0052 | 0.0259 | 0.9741 | 81356 | 2103 | 404639 | 3064385 | 37.7 | 0.0055 | 451 | 28952 | 0.0011 |
|  |  |  |  |  |  |  |  |  |  |  |  |  |  |  |  |  |
| 50-54 | 21160 | 170 | 56 | 2.680 | 0.0080 | 0.0394 | 0.9606 | 79253 | 3125 | 391180 | 2659746 | 33.6 | 0.0130 | 1030 | 28501 | 0.0026 |
|  |  |  |  |  |  |  |  |  |  |  |  |  |  |  |  |  |
| 55-59 | 15957 | 173 | 76 | 2.621 | 0.0108 | 0.0528 | 0.9472 | 76128 | 4023 | 373278 | 2268566 | 29.8 | 0.0232 | 1767 | 27472 | 0.0048 |
|  |  |  |  |  |  |  |  |  |  |  |  |  |  |  |  |  |
| 60-64 | 11914 | 166 | 74 | 2.623 | 0.0139 | 0.0674 | 0.9326 | 72105 | 4862 | 353445 | 1895288 | 26.3 | 0.0301 | 2167 | 25704 | 0.0062 |
|  |  |  |  |  |  |  |  |  |  |  |  |  |  |  |  |  |

| **Table 5 CONTINUATION** | | | | | | | | | | | | | | | | |
| --- | --- | --- | --- | --- | --- | --- | --- | --- | --- | --- | --- | --- | --- | --- | --- | --- |
| Age x | PYO | D^All^ | D^NCD^ | _n_a_x_ | _n_m_x_ | _n_q_x_ | _n_p_x_ | l_x_ | _n_d_x_ | _n_L_x_ | T_x_ | e_x_ | _n_q_x_^NCD^ | _n_d_x_ ^NCD^ | l_x_ ^NCD^ | _n_m_x_^NCD^ |
| 65-69 | 8959 | 182 | 78 | 2.644 | 0.0203 | 0.0969 | 0.9031 | 67242 | 6518 | 325287 | 1541843 | 22.9 | 0.0415 | 2793 | 23537 | 0.0087 |
|  |  |  |  |  |  |  |  |  |  |  |  |  |  |  |  |  |
| 70-74 | 7053 | 206 | 67 | 2.613 | 0.0292 | 0.1365 | 0.8635 | 60725 | 8290 | 285929 | 1216556 | 20.0 | 0.0444 | 2696 | 20743 | 0.0095 |
|  |  |  |  |  |  |  |  |  |  |  |  |  |  |  |  |  |
| 75-79 | 4949 | 187 | 73 | 2.615 | 0.0378 | 0.1733 | 0.8267 | 52434 | 9086 | 241230 | 930627 | 17.7 | 0.0676 | 3547 | 18047 | 0.0147 |
|  |  |  |  |  |  |  |  |  |  |  |  |  |  |  |  |  |
| 80-84 | 3311 | 176 | 62 | 2.418 | 0.0532 | 0.2337 | 0.7663 | 43348 | 10130 | 342145 | 689397 | 15.9 | 0.0823 | 3569 | 14500 | 0.0187 |
|  |  |  |  |  |  |  |  |  |  |  |  |  |  |  |  |  |
| 85+ | 4098 | 392 | 129 | 5.300 | 0.0957 | 1.0000 | 0.0000 | 33218 | 33218 | 347253 | 347253 | 10.5 | 0.3291 | 10931 | 10931 | 0.0315 |
|  |  |  |  |  |  |  |  |  |  |  |  |  |  |  |  |  |
| Total | 693,119 | 4,619 | 1,072 | - | - | - | - | - | - | - | - | - | - | 32,031 | - | - |

**Source: Kintampo HDSS (2005-2014)**

**Notes**

Age x = Age interval.

PYO = Person years of observation.

D^All^ _= All deaths attributable to the cohort.

D^NCD^_= Deaths attributable to non-communicable diseases.

_n_a_x_ = Average number of person-years lived in the interval by those who have died in the interval.

_n_m_x_ = Mortality rate for people in age group x to x + n.

_n_q_x_ = Probability of dying between ages x and x + n.

_n_p_x_ = Probability of surviving between ages x and x + n.

l_x_ = Number surviving at each age.

_n_d_x_ = Number of deaths between ages x and x + n.

_n_L_x_ = Person-years lived between ages x and x + n.

T_x_ = Person-years lived beyond age x.

e_x_ = Life expectancy at age x.

_n_q_x_^NCD^ = Probability of dying from non-communicable diseases between ages x and x + n.

_n_d_x_ ^NCD^ = Number of deaths from non-communicable diseases between ages x and x + n.

l_x_ ^NCD^ = Number surviving from non-communicable diseases at each age.

_n_m_x_^NCD^ = Mortality rate for non-communicable diseases from sage group x to x + n
