# Supplementary material for: A Competing Risk Analysis of Women Dying of Maternal, Infectious, or Non-Communicable Causes in the Kintampo Area of Ghana
Source: Front Glob Womens Health. 2021 Jun 21;2:690870. doi: 10.3389/fgwh.2021.690870 (PMC8593997; doi:10.3389/fgwh.2021.690870)
Supplement: Supplementary file 5 [file Data_Sheet_5.docx]

**Appendix V: Associated single-decrement life table for causes of death other than non-communicable diseases for Kintampo HDSS from 2005 to 2014**

| Age x | l_x_ | _n_p_x_ | R^-NCD^ | P^-NCD^ | l_x_^-NCD^ | _n_q_x_^-NCD^ | _n_d_x_^-NCD^ | _n_q_x_/ _n_q_x_^-NCD^ | _n_a_x_^-NCD^ | _n_m_x_^-NCD^ | _n_L_x_^-NCD^ | T_x_^-NCD^ | e_x_^-NCD^ |
| --- | --- | --- | --- | --- | --- | --- | --- | --- | --- | --- | --- | --- | --- |
| <1 | 100000 | 0.9567 | 0.9003 | 0.9609 | 100000 | 0.0391 | 3909 | 1.1083 | 0.4967 | 0.0399 | 98033 | 7711207 | 77.1 |
| 1-4 | 95668 | 0.9747 | 0.9004 | 0.9772 | 96091 | 0.0228 | 2195 | 1.1092 | 2.2530 | 0.0058 | 380531 | 7613174 | 79.2 |
| 5-9 | 93245 | 0.9919 | 0.8616 | 0.9930 | 93897 | 0.0070 | 656 | 1.1599 | 2.4971 | 0.0014 | 467842 | 7232643 | 77.0 |
| 10-14 | 92489 | 0.9930 | 0.6774 | 0.9952 | 93241 | 0.0048 | 443 | 1.4745 | 2.4657 | 0.0010 | 465081 | 6764801 | 72.6 |
| 15-19 | 91841 | 0.9918 | 0.7672 | 0.9937 | 92798 | 0.0063 | 583 | 1.3021 | 2.6986 | 0.0013 | 462647 | 6299720 | 67.9 |
| 20-24 | 91090 | 0.9865 | 0.8037 | 0.9892 | 92215 | 0.0108 | 999 | 1.2426 | 2.6863 | 0.0022 | 458763 | 5837072 | 63.3 |
| 25-29 | 89864 | 0.9806 | 0.8310 | 0.9838 | 91216 | 0.0162 | 1476 | 1.2418 | 2.6017 | 0.0034 | 452540 | 5378309 | 59.0 |
| 30-34 | 88117 | 0.9764 | 0.8108 | 0.9808 | 89740 | 0.0192 | 1719 | 1.2361 | 2.5583 | 0.0039 | 444501 | 4925769 | 54.9 |
| 35-39 | 86039 | 0.9716 | 0.7797 | 0.9778 | 88021 | 0.0222 | 1958 | 1.2674 | 2.5072 | 0.0045 | 435223 | 4481268 | 50.9 |
| 40-44 | 83593 | 0.9740 | 0.7978 | 0.9792 | 86063 | 0.0208 | 1787 | 1.2642 | 2.4705 | 0.0042 | 425795 | 4046045 | 47.0 |
| 45-49 | 81423 | 0.9743 | 0.7857 | 0.9798 | 84276 | 0.0202 | 1705 | 1.2782 | 2.5502 | 0.0041 | 417205 | 3620250 | 43.0 |
| 50-54 | 79333 | 0.9606 | 0.6706 | 0.9734 | 82572 | 0.0266 | 2198 | 1.4814 | 2.5668 | 0.0054 | 407510 | 3203045 | 38.8 |
| 55-59 | 76204 | 0.9472 | 0.5607 | 0.9700 | 80374 | 0.0300 | 2410 | 1.7625 | 2.5658 | 0.0061 | 396002 | 2795535 | 34.8 |
| 60-64 | 72177 | 0.9326 | 0.5542 | 0.9620 | 77964 | 0.0380 | 2959 | 1.7767 | 2.6292 | 0.0077 | 382804 | 2399532 | 30.8 |
| 65-69 | 67310 | 0.9031 | 0.5714 | 0.9434 | 75005 | 0.0566 | 4245 | 1.7127 | 2.6823 | 0.0116 | 365186 | 2016728 | 26.9 |
| 70-74 | 60786 | 0.8635 | 0.6748 | 0.9057 | 70760 | 0.0943 | 6673 | 1.4478 | 2.5866 | 0.0197 | 337696 | 1651543 | 23.3 |
| 75-79 | 52487 | 0.8267 | 0.6096 | 0.8905 | 64087 | 0.1095 | 7020 | 1.5820 | 2.5702 | 0.0230 | 303379 | 1313847 | 20.5 |
| 80-84 | 43392 | 0.7663 | 0.6477 | 0.8416 | 57067 | 0.1584 | 9037 | 1.4757 | 2.4283 | 0.0344 | 262096 | 1010467 | 17.7 |
| 85+ | 33251 | 0.0000 | 0.6709 | 0.0000 | 48030 | 1.0000 | 48030 | 1.0000 | 15.5813 | 0.0642 | 748372 | 748372 | 15.6 |

**Source: Kintampo HDSS (2005-2014)**

**Note**:

Age x = Age interval.

l_x_ = Number surviving at each age.

_n_p_x_ = Probability of surviving between ages x and x + n.

R^− NCD^ = the proportion of deaths due to all causes other than non-communicable diseases.

P^- NCD^ = Probability of surviving all causes of deaths other than non-communicable diseases.

l_x_^- NCD^ = Number surviving at each age from all causes of deaths other than non-communicable diseases.

_n_q_x_^- NCD^ = Probability of dying from all causes of deaths other than non-communicable diseases between ages x and x + n.

_n_d_x_^- NCD^ = Number of deaths from all causes of deaths other than non-communicable diseases between ages x and x + n.

_n_q_x_/ _n_q_x_^- NCD^ = Probability of dying between ages x and x + n divided by probability of dying from all causes of deaths other than non-communicable diseases between ages x and x + n.

_n_a_x_^- NCD^ = Average number of person-years lived in the interval by those who have died in the interval from all causes other than non-communicable diseases.

_n_m_x_^- NCD^ = Mortality rate for people in age group x to x + n from all causes of deaths other than non-communicable diseases.

_n_L_x_^- NCD^ = Person-years lived between ages x and x + n from all causes of deaths other than non-communicable diseases.

T_x_^- NCD^ = Person-years lived beyond age x from all causes of deaths other than non-communicable diseases.

e_x_^- NCD^ = Life expectancy at age x from all causes of deaths other than non-communicable diseases.
